# Leptospirosis Health Intervention Module Effect on Knowledge, Attitude, Belief, and Practice among Wet Market Workers in Northeastern Malaysia: An Intervention Study

**DOI:** 10.3390/ijerph15071396

**Published:** 2018-07-03

**Authors:** Mas Harithulfadhli Agus Ab Rahman, Suhaily Mohd Hairon, Rukman Awang Hamat, Tengku Zetty Maztura Tengku Jamaluddin, Mohd Nazri Shafei, Norazlin Idris, Malina Osman, Surianti Sukeri, Zainudin A. Wahab, Wan Mohd Zahiruddin Wan Mohammad, Zawaha Idris, Aziah Daud

**Affiliations:** 1 Department of Community Medicine, School of Medical Sciences, Universiti Sains Malaysia, Kubang Kerian 16150, Malaysia; masharithul@outlook.com (M.H.A.A.R.); suhailymh@usm.my (S.M.H.); drnazri@usm.my (M.N.S.); norazlin@usm.my (N.I.); surianti@usm.my (S.S.); drzahir@usm.my (W.M.Z.W.M.); 2 Department of Medical Microbiology and Parasitology, Faculty of Medicine and Health Sciences, Universiti Putra Malaysia (UPM), Serdang 43400, Malaysia; rukman@upm.edu.my (R.A.H.); tengkuzetty@upm.edu.my (T.Z.M.T.J.); malinaosman@upm.edu.my (M.O.); 3 Health Department of Federal Territory Kuala Lumpur and Putrajaya, Jalan Cenderasari, Kuala Lumpur 50590, Malaysia; zwah58@gmail.com; 4 Health Promotion Unit, Penang State Health Department, Floor 7, Bangunan Persekutuan, Jalan Anson 10400, Penang, Malaysia; hjhzawaha@yahoo.com

**Keywords:** leptospirosis, wet market workers, intervention, health education

## Abstract

Leptospirosis is an infectious disease which can be prevented by increasing awareness and promoting preventive health behaviours among high risk groups including wet market workers. Thus, the aim of this study was to determine the effectiveness of Leptospirosis Health Intervention Program (LHIP) in improving knowledge, attitude, belief and practice towards leptospirosis among wet market workers in Kelantan. This intervention study was conducted in two main wet markets in Kelantan involving 116 participants in each control and intervention groups. The health education intervention was based on Leptospirosis Health Intervention Module. The knowledge, attitude, belief and practice scores were measured before and six weeks after the intervention to examine the effect of the program. The results showed that knowledge (*p* < 0.001), attitude (*p* = 0.001), belief (*p* < 0.001) and practice (*p* < 0.001) scores changes were significantly higher in the intervention group compared to the control group. The adjusted mean differences were 12.93 (95% CI: 8.47, 17.39) for knowledge, 5.55 (95% CI: 2.28, 8.81) for attitude, 7.21 (95% CI: 3.43, 10.99) for belief and 7.35 (95% CI: 3.64, 11.05) for practice scores. Leptospirosis Health Intervention Program was an effective health educational tools to improve awareness and preventive behaviours among risk groups such as wet market workers.

## 1. Introduction

Leptospirosis is caused by bacteria from the genus *Leptospira*. It is a gram-negative bacteria that is thin and helically coiled in shape [1,2,3]. The bacteria can be categorized into pathogenic and saprophytic groups. More than 250 leptospira serotypes have been recognized within the pathogenic group which can cause disease in human [4]. Leptospirosis is a zoonotic disease, which means the bacteria are carried by animals before it is transmitted to human. Both wild and domestic animals can be carriers for the bacteria, and the main animal reservoir for human infection globally are rodents. These animals excrete leptospira through urine during their lifetime and contaminate the environment [5,6]. Humans can become infected with leptospirosis through contact with the environments contaminated with urine of infected animals. Infected humans develop a spectrum of symptoms that can mimic other febrile illnesses including dengue, malaria, and typhoid [7].

Globally, leptospirosis occurs in diverse geographical settings due to the large spectrum of animal hosts that can carry the pathogen in their renal tubules. However, the incidence of leptospirosis is higher in humid and warm countries where survival of leptospira in environment are favourable. It is estimated that the number of leptospirosis exceed one million cases every year around the globe. The number of deaths due to leptospirosis is estimated at 58,900 cases each year. The estimated incidence of leptospirosis was 14.77 cases per 100,000 population, and the mortality incidence due to leptospirosis was estimated at 0.84 deaths per 100,000 population worldwide [8]. Within certain risk groups, the incidence of leptospirosis is higher due to the increased exposure to contaminated environments [7]. Leptospirosis has been recognized as a hazard in certain occupations with increased exposure to infected animals such as agricultural workers, sewage workers, military personnel, veterinarians, and animal handlers [9,10].

A wet market is an open food market. Workers at these markets involved with activities of selling fresh meat, fish, fruits, vegetables and dried processed foods. Wet market workers are at risk for leptospirosis due to exposure to contaminated environments at their workplace. Humid conditions and abundant food supply are suitable for rodents’ infestation, which is the main reservoir for human leptospirosis. Previous studies have documented the risk of exposure at market areas [11,12,13]. An effective tool is needed to increase awareness and improve preventive behaviours among wet market workers. Thus, the aim of this study was to determine the effectiveness of Leptospirosis Health Intervention Program in improving knowledge, attitude, belief, and practice toward leptospirosis among wet market workers in Kelantan, Malaysia.

## 2. Materials and Methods

An intervention study was conducted in two main wet markets in Kelantan which are located in the north-eastern part of Peninsular Malaysia. Siti Khadijah Market and Pasir Mas Market are located in Kota Bharu and Pasir Mas districts. Kota Bharu and Pasir Mas districts recorded the highest number of leptospirosis cases in 2014 [14]. This study was conducted from January 2017 to June 2017. A total of 116 workers from each wet market were randomly selected to participate in this study. The criteria for the eligible participants were age of 18 years and above and worked for at least three months in the markets. The participants from Siti Khadijah Market were assigned to intervention group and participants from Pasir Mas Market were assigned to control group. The assignment of participants to intervention and control groups based on work locations were made to avoid contamination of intervention program given to the intervention group.

### 2.1. Knowledge, Attitude, Belief, Practice Questionnaire

A validated questionnaire on knowledge, attitude, belief and practice (KABP) regarding leptospirosis was used to collect pre-intervention and post-intervention data from the participants [15]. The questionnaire consisted of two parts; The general information section and KABP section. General information section collects information on sociodemographic data of participants and this information were self-reported by the participants. The knowledge section consisted of questions regarding leptospirosis on causative agent, transmission of disease, diagnostic tests, symptoms, complications and preventive measures. There were three answer options of “true”, “false” and “unsure”. The correct answer was scored as “1”, whereas incorrect or unsure answers were scored as “0”. The attitude section consisted of questions on attitude regarding hygienic practices, control and prevention practices, avoidance of exposures, health seeking behaviours and usage of personal protective equipment. The answer options were “strongly agree”, “agree”, “unsure”, “disagree”, to “strongly disagree”. The attitude score was recorded from 1 to 5. The belief section consisted of questions on belief regarding benefit, barrier and self-efficacy aspects on leptospirosis. There were five items in belief section with five Likert-scale options from “strongly agree”, “agree”, “unsure”, “disagree”, to “strongly disagree”. The score was recorded from 1 to 5. The practice section consisted of questions on preventive and risk reduction practices on leptospirosis. There were 17 items in practice section with five Likert-scale options from “always”, “most of the time”, “seldom”, “never”, to “not related”. The score was recorded from 0 to 4. Scores from KABP section were calculated for overall percentage for each knowledge, attitude, belief and practice section.

### 2.2. Leptospirosis Health Intervention Program (LHIP)

The program was based on Leptospirosis Health Intervention Module (LHIM) which was developed by a panel of experts including epidemiologists, occupational health specialists, microbiologists, health educationists, and medical statisticians. The LHIM was developed following extensive literature reviews and serial discussions among the experts to ensure good content validity and relevancy of information regarding leptospirosis. The module consisted of four scopes on leptospirosis and various activities that covered topics on introduction to leptospirosis, diagnosis and treatment, risk for infection, prevention, and control measures. Various methods were used to deliver the contents of the LHIM including lectures, video presentations, games, roleplay, small group discussions, demonstrations, and hands-on. The lectures and activities during the program were carried out by experts and trained staffs whom also involved in developing the module. The intervention program was conducted in January 2017, and all participants in intervention group received similar intervention program based on LHIP. 

### 2.3. Data Collection

Ethical clearance was obtained from Research and Ethic Committee (Human), School of Medical Sciences, Health Campus, Universiti Sains Malaysia (USM/JEPeM/15120552). The study was explained in sufficient detail, and written consent was obtained from all participants before the conduct of the study. Prior to data collection, co-researchers were trained regarding the KABP questionnaire to reduce interrater bias. The researcher and co-researchers used a face-to-face interview guided method to obtain information from the participants. The pre-intervention KABP data collection was conducted prior to the intervention program. The intervention program was conducted among participants in the intervention group. Meanwhile, the participants in the control group received no intervention program. Post-intervention data collection was carried out six weeks after the intervention program.

### 2.4. Data Analysis

Data were analysed using IBM SPSS statistics version 24.0 (IBM, Armonk, NY, USA). Numerical variables were presented as means and standard deviations (SD) whereas categorical data were presented as frequencies and percentages. To determine the effect of Leptospirosis Health Intervention Program, multi-way ANOVA was performed to compare the pre-intervention and post-intervention changes in knowledge, attitude, belief, and practice scores between the intervention and control groups.

## 3. Results

### 3.1. Sociodemographic Characteristics of Respondents in Control and Intervention Groups

During the initial stage of this study, 232 participants were selected from Siti Khadijah and Pasir Mas Market, 116 from each market. However, only 88 participants from the control group and 82 participants from the intervention groups completed the study. Table 1 shows the sociodemographic characteristics of respondents in the control and intervention groups. The mean age of respondents in the intervention group was slightly higher than the control group. All respondents were Malay. The intervention group had a higher proportion of female participants in comparison to the control group. Majority of respondents were married and attained secondary school education.

### 3.2. Knowledge Section

Table 2 shows the descriptive statistics of pre-intervention and post-intervention knowledge scores. The effect of LHIP intervention after adjusting for the effect of gender and monthly income is presented in Table 3. The adjusted mean knowledge score changes for the control and intervention groups were 3.60 and 16.54 respectively. The adjusted mean difference was 12.93 (95% CI 8.47, 17.39). Those in the intervention group shows significantly higher scores compared to the control group (*p* < 0.001). Gender and monthly income were not significant factors for mean knowledge score changes. Multi-way ANOVA analysis showed that there was no significant interaction among groups and gender (F(1, 164) = 0.99, *p* = 0.320) and groups and monthly income (F(2, 163) = 0.60, *p* = 0.548) on knowledge score changes.

### 3.3. Attitude Section

Table 4 shows the descriptive statistics of pre-intervention and post-intervention attitude scores. The effect of LHIP intervention after adjusting for the effect of gender and monthly income is presented in Table 5. The adjusted mean attitude score changes for the control and intervention groups were −1.95 and 3.59 respectively. The adjusted mean difference was 5.55 (95% CI 2.28, 8.81). Those in the intervention group shows significantly higher scores compared to the control group (*p* = 0.001). Gender and monthly income were not significant factors for mean attitude score changes. Multi-way ANOVA analysis showed that there was no significant interaction among groups and gender (F(1, 164) = 0.11, *p* = 0.733) on attitude score changes. There was significant interaction among groups and monthly income (F(2, 163) = 5.05, *p* = 0.007) on attitude score changes.

### 3.4. Belief Section

Table 6 shows the descriptive statistics of pre-intervention and post-intervention belief scores. The effect of LHIP intervention after adjusting for the effect of gender and monthly income is presented in Table 7. The adjusted mean belief score changes for control and intervention groups were −1.39 and 5.82 respectively. The adjusted mean difference was 7.21 (95% CI 3.43, 10.99). Those in the intervention group shows significantly higher scores compared to the control group (*p* < 0.001). Gender and monthly income were not significant factors for mean belief score changes. Multi-way ANOVA analysis showed that there were no significant interaction among groups and gender (F(1, 164) = 0.51, *p* = 0.473) and groups and monthly income (F(2, 163) = 2.91, *p* = 0.057) on belief score changes.

### 3.5. Practice Section

Table 8 shows the descriptive statistics of pre-intervention and post-intervention practice scores. The effect of LHIP intervention after adjusting for the effect of gender and monthly income is presented in Table 9. The adjusted mean practice score changes for the control and intervention groups were 1.06 and 8.41 respectively. The adjusted mean difference was 7.35 (95% CI 3.64, 11.05). Those in the intervention group shows significantly higher scores compared to the control group (*p* < 0.001). Gender and monthly income were not significant factors for mean practice score changes. Multi-way ANOVA analysis showed that there were no significant interaction among groups and gender (F(1, 162) = 0.19, *p* = 0.659) and groups and monthly income (F(2, 161) = 0.19, *p* = 0.823) on practice score changes.

## 4. Discussion

Leptospirosis is a zoonotic disease affects human who have direct or indirect contact with the urine of infected animals. The disease can be prevented and controlled using strategies focusing on controlling the source of infection, interrupting route of transmission, and prevention at the human level. The source of infection can be controlled by measures such as a reducing reservoir animal population, separation of human from animal habitats, and immunization of pets and livestock. Transmission of infection can be interrupted by minimizing contact with polluted environments such as using protective clothing and covering wounds where exposure is expected. Human can be protected from infection and disease by increasing awareness regarding leptospirosis among the public, especially those involved in high-risk activities. The public can protect themselves from infection by taking necessary measures and recognizing the disease at early stage to get treatment. Vaccines for humans are available but the use of vaccination in the population is limited due to serovars-specific protection provided by the vaccines [7].

The Leptospirosis Health Intervention Module (LHIM) was developed as a health educational module to increase awareness regarding leptospirosis among the public especially risk groups. Health education is defined as “activities which raise an individual’s awareness, giving the individual the health knowledge required to enable him or her to decide on a particular health action” [16]. This is to empower the public to practice preventive measures in line with the WHO target for health promotion [17]. Specifically, the aim of LHIM is to improved knowledge, attitude, belief and practice regarding prevention and control of leptospirosis among risk groups.

The changes in knowledge, attitude, belief and health practice among respondents in this study can be explained using Health Belief Model (HBM) theory. HBM theory explains that actions toward health are influenced by how people perceived susceptibility to a disease, severity of the disease, and benefits of preventive actions outweigh the barriers toward actions and self-efficacy [18]. Perceived susceptibility means that the people believe they are exposed and can contract the disease, while perceived severity refers to the consequences of having the disease including clinical and social effects. Both perceived susceptibility and perceived severity are known as perceived threat. For action to be taken, the decision is influenced by perceived benefits, which are beliefs regarding the benefits of the actions that can be taken. Perceived barriers are negative aspect of the actions such as cost, side effects, and time spent to do the action. People also need to believe that they can successfully do the required actions to prevent the disease which is the perceived self-efficacy.

### 4.1. Effect of Leptospirosis Health Intervention Program on Knowledge

The results in this study shows that LHIP intervention given to intervention group were able to increase the knowledge of the respondents. The mean score of knowledge significantly increase 3.59 points in intervention group compared to −1.95 points in control group after six weeks post-intervention. In the module of LHIM, respondents in intervention group were educated regarding information on leptospirosis which include the aetiological agent, carrier animals, transmission of the disease, local distribution of the disease, risk groups and risk areas of leptospirosis, symptoms, clinical staging, and severity of the disease. The respondents were also introduced to simple preventive measures that can be taken to avoid infection. These measures include hand washing technique, proper use of personal protective equipment, wound covering during risk activities, and hygienic practices at home and in the workplace. The barriers to taking preventive steps were also discussed during the intervention program. For example, respondents were informed regarding various types of gloves and mask available in the market that were affordable. Simple measures such as hygienic care, hand wash practice, and wound care can prevent leptospirosis infection.

The contents of the LHIP intervention, which was developed by specialists from various fields, were adequate to educate the respondents in this study regarding basic knowledge about leptospirosis. The contents cover all the aspects of Health Belief Model theory of behavioural changes including perceived susceptibility, perceived severity, perceived benefits, perceived barriers, and perceived self-efficacy. The LHIM was developed in Malay language using simple terms to convey medical information to the population. This is important as the majority of Malaysian especially in Kelantan speak in Malay language.

Various methods for delivering health education contents were used in the intervention program. Lectures, video presentations, role play, small groups discussions, demonstrations and games were used as a medium to convey the messages. Each method has its own advantages and limitations. For example, lecture is effective at presenting fact materials to large groups of people, but it is a one-way communication and the degree of acceptance is difficult to measure. Video presentation is an entertaining educational session. It can supplement the content of a lecture, but it has similar limitations as a lecture. As for small groups discussion, it allows everyone to participate and give their opinions on the subject. However, good facilitator is needed as the discussion can get side tracked. Demonstrations such as hand washing technique and proper use of personal protective equipment can give a better understanding of practical preventive actions compare to lecture or video presentation. However, demonstration is more effective in small group compared to large audience [19]. By combining multiple methods in the LHIM program, the delivery of health message can be more effective.

As a comparison, an intervention study by Azfar et al. [20] using the Leptospirosis Interactive Health Promotion Modul (LIHPM) significantly improved the knowledge score of respondents in the intervention group. The study was conducted among town service workers in Kelantan. In the study, the LIHPM was a two days program which includes animation show, interview, mind mapping, practical session, games, and roleplay. Similarly, an educational intervention study by Bipin et al. [21] was conducted in Navsari district, India. Educational messages regarding leptospirosis were given to residents of villages in the district using street plays and poster exhibition. The street plays were performed twice in the villages followed by poster presentation regarding cause, transmission, symptoms and measures to prevent leptospirosis. The study found that the knowledge of the residents was significantly increased after the intervention. The researchers suggested that educational intervention such as plays and posters in local language can be an effective tools to increase awareness in community [21].

### 4.2. Effect of Leptospirosis Health Intervention Program on Attitude

Attitude is defined as “a feeling or opinion about something or someone, or a way of behaving” [22]. In this study, the effect of LHIM on knowledge, attitude, belief and practice regarding leptospirosis was evaluated. The attitude of risk groups toward leptospirosis was investigated in previous studies [20,23]. Azfar et al. [20] reported that 48.0% of respondents had unsatisfactory attitude towards leptospirosis among town service workers in Kelantan. The study also found that the positive attitude at workplace was lower than positive attitude during off work. In another study by Arbiol et al. [23] on knowledge, attitude and practice toward leptospirosis among Communities of Calamba and Los Banos, Philippines found that the attitude score toward leptospirosis was higher compared to knowledge and practice score among the respondents. The researchers proposed that attitude alone is not adequate to transform to good health practices and need to be complemented by sufficient knowledge to be translated to good preventive actions [23]. A study by Azfar et al. [20] showed that with effective intervention program, knowledge regarding the disease improved which also lead to improvement of attitude among the town service workers. In the study, the attitude score of intervention group increased significantly compared to control group after six weeks of intervention. The study demonstrated an increase of attitude score from 66.02 to 93.36 in intervention group. 

In this current study, there was a significant increase in attitude score among respondents in intervention group compared to the control group. This change was attributed to the increase of knowledge after the intervention program. The knowledge score regarding leptospirosis increased significantly in intervention group which indicate the LHIP were able to successfully convey the health messages to respondents. With better understanding regarding aetiology, transmission, risk factors, and severity of the disease, the attitude score of respondents toward leptospirosis improved. The finding in this study indicate that attitude of the workers toward leptospirosis is influenced by their knowledge regarding the disease [24,25].

### 4.3. Effect of Leptospirosis Health Intervention Program on Belief

In this study, the belief score increased significantly among the intervention group compared to control group after six weeks of intervention program. The belief score regarding leptospirosis among wet market workers increased from 83.51 to 90.00 in intervention group compared to a decreased from 85.86 to 84.55 in control group. This result showed that the belief regarding leptospirosis was congruence with the knowledge and attitude of the workers toward leptospirosis. Evidence from literature suggested that retrieval, formation and modification in beliefs are influenced by attitudes [26]. With relevant knowledge given to the workers regarding threat (susceptibility and severity) of leptospirosis and measures for prevention and control of the disease through the LHIP intervention program, the attitude and belief of the respondents can be improved. To our best knowledge, there were lack of literatures on evaluation of belief domain in relation to effect of health education on leptospirosis. Thus, direct comparisons were difficult.

However, a study on educational program among glaucoma patients by Mohamed et al. [27] demonstrated that the intervention improved belief regarding incorrect cause of glaucoma. The study used educational program content that include information regarding glaucoma, misconceptions on glaucoma, and demonstrations on using eyedrop and eye exercise. The program used local language to deliver the health message. The researchers found that the knowledge, attitude, belief, and practice regarding glaucoma improved significantly after the intervention program. These findings showed that good educational health intervention can increased knowledge and improved attitude and belief of the respondents.

### 4.4. Effect of Leptospirosis Health Intervention Program on Practice

The results in this study showed a significant improvement in overall practice score among respondents in the intervention group compare to the control group. The practice score increased from 76.81 to 86.03 in intervention group compared to 77.07 to 78.28 in control group. The LHIP intervention program incorporated the knowledge regarding risk activities that exposed to leptospirosis with measure for prevention and control that should be taken to reduce risk of exposure and infection to the disease. These include hygienic practice at home and in the workplace, the practice of managing garbage, the practice of using of personal protective equipment, the practice of seeking medical treatment, and the practice of covering wounds in risk activities. Demonstration regarding hand washing technique and various use of PPE including masks, gloves, boots, and long sleeves were integrated into the intervention program. These activities increase awareness of respondents to healthy preventive practice toward leptospirosis. Improvement of knowledge, attitude, and belief of the workers resulted in improvement of the practice score.

The significant improvement in overall mean score of practice this study was similar to study by Azfar et al. [20]. In the study, the researchers found that there was significant difference in practice score between intervention and control group of the town service workers after the intervention program. The mean practice score increased from 58.81 to 85.55 and decreased from 60.19 to 59.75 in intervention and control groups respectively. Similarly, the intervention program used in the study included activities such as personal protective equipment hands on, hand washing technique with soap, hand rub technique with sanitizer, and roleplay [20]. This supported the evidence that effective health education program can promote positive health behavior. Positive attitude and belief complemented with relevant knowledge will improve the individuals ability to translate prevention measures into action [23]. These findings were also supported by studies on other infectious diseases. Significant association were demonstrated between knowledge, attitude, and health-related behaviour in studies on dengue and rabies [28,29,30]. This evidence puts emphasis on the education as an important tool to improve knowledge, attitude, and prevention practices against leptospirosis among risk groups.

## 5. Conclusions

In conclusion, the Leptospirosis Health Intervention Program was proven to effectively improve knowledge, attitude, belief, and practice scores on leptospirosis among wet market workers. This tool can be used for health education among risk groups especially wet market workers to improve their awareness regarding leptospirosis and their preventive practices against the disease.

## Figures and Tables

**Table 1:** Sociodemographic characteristics of respondents in intervention and control group (*n* = 170).

Variables	Frequency (%)		*p*-Value
	Control Group *n* = 88	Intervention Group *n* = 82	
Age	43.90 (13.84) ^a^	44.98 (14.89) ^a^	0.625 ^c^
Duration of work (month)	84 (200) ^b^	114 (182) ^b^	0.368 ^d^
Gender			0.030 ^e^
Male Female	34 (38.6) 54 (61.4)	19 (23.2) 63 (76.8)	
Marital status			
Single/widower Married	14 (15.9) 74 (84.1)	23 (28.0) 59 (72.0)	0.055 ^e^
Monthly income (RM)			
0–580 581–940 >940	32 (36.4) 24 (27.3) 32 (36.4)	15 (18.3) 32 (39.0) 35 (42.7)	0.027 ^e^
Educational level			
No formal education Primary school Secondary school Form 6/Higher education	9 (10.2) 10 (11.4) 56 (63.6) 13 (14.8)	6 (7.3) 10 (12.2) 47 (57.3) 19 (23.2)	0.512 ^e^

^a^ Mean (SD); ^b^ Median (IQR); ^c^ Independent *T*-test; ^d^ Mann-Whitney test; ^e^ Chi-square.

**Table 2:** Descriptive statistics of pre-intervention and post-intervention knowledge score.

Variable	Mean (SD)	
	Preintervention	Postintervention
Control Intervention	78.65 (13.19) 75.20 (13.29)	82.15 (13.13) 92.07 (8.68)

**Table 3:** Effect of intervention on pre-post mean knowledge score changes by adjusting for gender and monthly income (*n* = 170).

Variable	Pre-Post Mean Score Difference		F-Stat (df)	*p*-Value
	Adj. Mean (95% CI) ^a^	Adj. Mean diff. (95% CI) ^b^		
Group Control Intervention	3.60 (0.57, 6.64) 16.54 (13.09, 19.99)	12.93 (8.47, 17.39)	32.82 (1)	<0.001
Gender Male Female	9.82 (5.87, 13.78) 10.32 (7.73, 12.91)	0.50 (−4.23, 5.23)	0.04 (1)	0.834
Monthly income (RM) 0–580 581–940 >940	9.03 (4.65, 13.41) 11.86 (8.06, 15.67) 9.33 (5.84, 12.81)	−2.83 (−9.78, 4.11) ^c^ 2.53 (−3.65, 8.72) ^d^ 0.30 (−6.34, 6.94) ^e^	0.65 (2)	0.521

No significant interaction between groups and gender (F(1, 164) = 0.99, *p* = 0.320); No significant interaction between groups and monthly income (F(2, 163) = 0.60, *p* = 0.548); ^a^ Adjusted means using Three-way ANOVA analysis; ^b^ Bonferroni adjustment for 95% CI for difference; ^c^ Mean for monthly income RM 0–580—mean for monthly income RM 581–940; ^d^ Mean for monthly income RM 581–940—mean for monthly income RM >940; ^e^ Mean for monthly income RM >940—mean for monthly income RM 0–580.

**Table 4:** Descriptive statistics of pre-intervention and post-intervention attitude score.

Variable	Mean (SD)	
	Preintervention	Postintervention
Control Intervention	88.27 (7.83) 87.37 (7.84)	86.68 (8.79) 92.17 (8.88)

**Table 5:** Effect of intervention on pre-post mean attitude score changes by adjusting for gender and monthly income (*n* = 170).

Variable	Pre-Post Mean Score Difference		F-Stat (df)	*p*-Value
	Adj. Mean (95% CI) ^a^	Adj. Mean diff. (95% CI) ^b^		
Group Control Intervention	−1.95 (−4.17, 0.26) 3.59 (1.06, 6.12)	5.55 (2.28, 8.81)	11.25 (1)	0.001
Gender Male Female	−0.75 (−3.65, 2.14) 2.40 (0.50, 4.30)	3.15 (−0.30, 6.62)	3.23 (1)	0.074
Monthly income (RM) 0–580 581–940 >940	−0.64 (−3.85, 2.56) 0.81 (−1.97, 3.59) 2.30 (−0.24, 4.85)	−1.45 (−6.54, 3.63) ^c^ −1.49 (−6.03, 3.04) ^d^ 2.95 (−1.91, 7.82) ^e^	1.09 (2)	0.337

No significant interaction between groups and gender (F(1, 164) = 0.11, *p* = 0.0.733); Significant interaction between groups and monthly income (F(2, 163) = 5.05, *p* = 0.007); ^a^ Adjusted means using Three-way ANOVA analysis; ^b^ Bonferroni adjustment for 95% CI for difference; ^c^ Mean for monthly income RM 0–580—mean for monthly income RM 581–940; ^d^ Mean for monthly income RM 581–940—mean for monthly income RM >940; ^e^ Mean for monthly income RM >940—mean for monthly income RM 0–580.

**Table 6:** Descriptive statistic of pre-intervention and post-intervention belief score.

Variable	Mean (SD)	
	Preintervention	Postintervention
Control Intervention	85.86 (8.72) 83.51 (9.14)	84.55 (10.97) 90.00 (10.04)

**Table 7:** Effect of intervention on pre-post mean belief score changes by adjusting for gender and monthly income (*n* = 170).

Variable	Pre-Post Mean Score Difference		F-Stat (df)	*p*-Value
	Adj. Mean (95% CI) ^a^	Adj. Mean diff. (95% CI) ^b^		
Group Control Intervention	−1.39 (−3.95, 1.17) 5.82 (2.90, 8.75)	7.21 (3.43, 10.99)	14.22 (1)	<0.001
Gender Male Female	1.86 (−1.48, 5.22) 2.56 (0.37, 4.76)	0.69 (−3.31, 4.71)	0.11 (1)	0.731
Monthly income (RM) 0–580 581–940 >940	0.26 (−3.44, 3.98) 2.30 (−0.92, 5.52) 4.08 (1.13, 7.04)	−2.03 (−7.92, 3.85) ^c^ −1.78, (−7.03, 3.46) ^d^ 3.81 (−1.81, 9.45) ^e^	1.35 (2)	0.260

No significant interaction between groups and gender (F(1, 164) = 0.51, *p* = 0.473); No significant interaction between groups and monthly income (F(2, 163) = 2.91, *p* = 0.057); ^a^ Adjusted means using Three-way ANOVA analysis; ^b^ Bonferroni adjustment for 95% CI for difference; ^c^ Mean for monthly income RM 0–580—mean for monthly income RM 581–940; ^d^ Mean for monthly income RM 581–940—mean for monthly income RM >940; ^e^ Mean for monthly income RM >940—mean for monthly income RM 0–580.

**Table 8:** Descriptive statistic of pre-intervention and post-intervention practice score.

Variable	Mean (SD)	
	Preintervention	Postintervention
Control Intervention	77.07 (10.32) 76.81 (9.77)	78.28 (12.81) 86.03 (8.93)

**Table 9:** Effect of intervention on pre-post mean practice score different by adjusting for gender and monthly income (*n* = 170).

Variable	Pre-Post Mean Score Difference		F-Stat (df)	*p*-Value
	Adj. Mean (95% CI) ^a^	Adj. Mean diff. (95% CI) ^b^		
Group Control Intervention	1.06 (−1.47, 3.61) 8.41 (5.55, 11.27)	7.35 (3.64, 11.05)	15.31 (1)	<0.001
Gender Male Female	3.84 (0.54, 7.14) 5.64 (3.48, 7.79)	1.79 (−2.14, 5.74)	0.81 (1)	0.369
Monthly income (RM) 0–580 581–940 >940	3.52 (−0.12, 7.17) 3.92 (0.73, 7.11) 6.78 (3.89, 9.66)	−0.39 (−6.19, 5.40) ^c^ −2.86 (−8.01, 2.29) ^d^ 3.25 (−2.27, 8.78.) ^e^	1.35 (2)	0.260

No significant interaction between groups and gender (F(1, 162) = 0.19, *p* = 0.659); No significant interaction between groups and monthly income (F(2, 161) = 0.19, *p* = 0.823); ^a^ Adjusted means using Three-way ANOVA analysis; ^b^ Bonferroni adjustment for 95% CI for difference; ^c^ Mean for monthly income RM 0–580—mean for monthly income RM 581–940; ^d^ Mean for monthly income RM 581–940—mean for monthly income RM >940; ^e^ Mean for monthly income RM >940—mean for monthly income RM 0–580.
